# Predictive equations commonly used in the clinic underestimate resting energy expenditure compared with whole-room indirect calorimetry in colorectal cancer survivors

**DOI:** 10.1016/j.ajcnut.2026.101209

**Published:** 2026-01-27

**Authors:** Rakel R Eklo, Dena T Alavi, Dina M Konglevoll, Åshild Kolle, Hege B Henriksen, Russell Rising, Rune Blomhoff, Thomas Olsen

**Affiliations:** 1 Department of Nutrition, Institute of Basic Medical Sciences, University of Oslo, Oslo, Norway; 2 D&S Consulting Services, Inc, Research & Developlement, New York, United States; 3 Department for Clinical Service, Division of Cancer Medicine, Oslo University Hospital, Oslo, Norway

**Keywords:** bioelectrical impedance analysis, body composition, colorectal cancer, colorectal cancer survivors, dual-energy X-ray absorptiometry, predictive equations, resting energy expenditure, respiratory quotient, whole-room indirect calorimetry

## Abstract

**Background:**

Accurate methods for estimating resting energy expenditure (REE) are important to ensure adequate nutritional treatment in colorectal cancer (CRC) survivors.

**Objectives:**

This study aims to determine the agreement between REE estimated by commonly used predictive equations and by whole-room indirect calorimetry (WRIC).

**Methods:**

This cross-sectional study included 31 CRC survivors {age: 53–78 y; mean [standard deviation (SD)]; body mass index 28.7 [4.28] kg/m^2^}, who underwent curative surgery. Body composition was measured by dual-energy X-ray absorptiometry (DXA). Predicted REE from equations in clinical use and derived from DXA and bioelectrical impedance analysis (BIA) were compared against REE measured by 30-min WRIC. Equations included Harris–Benedict, Mifflin–St. Jeor, Food and Agriculture Organization (FAO)/World Health Organization (WHO)/United Nations University (UNU), Henry, Mifflin–St. Jeor_DXA,_ and FAO/WHO/UNU_BIA_. Paired sample *t*-test, Lin’s concordance correlation coefficient, and Bland–Altman analysis were used to determine the agreement between measured REE_WRIC_ and predicted REE. Accuracy was defined as the percentage of predicted REE values that fell within ± 10% of REE_WRIC_.

**Results:**

Mean (SD) REE_WRIC_ was 1710 kcal/d (353), and respiratory quotient was 0.79 (0.05). Most equations underestimated REE. Overall, Harris–Benedict, Henry, and FAO/WHO/UNU_BIA_ showed the best overall agreement with REE_WRIC_. However, these equations showed low accuracy with 65%, 68%, and 62% of predicted REE values within ± 10% of REE_WRIC_, respectively.

**Conclusions:**

Most predictive equations tended to underestimate REE in CRC survivors compared with REE_WRIC_. The Harris–Benedict, Henry and FAO/WHO/UNU_BIA_ equations showed both best accuracy and agreement with WRIC. They were still inaccurate, with individual variability for a relevant part of the sample. Future studies need to develop improved predictive equations for CRC survivors. This study was registered at clinicaltrials.gov as NCT01570010 (https://clinicaltrials.gov/study/NCT01570010?locStr=Norway&country=Norway&cond).

## Introduction

Colorectal cancer (CRC) is the second deadliest of all cancers with ∼0.9 million global deaths in 2022 [1,2]. The number of CRC survivors [defined as individuals who have been diagnosed with cancer, starting from the time of diagnosis through the rest of their lives (World Cancer Research Fund, 2025)] is rising due to increasing incidence and improved survival [3,4]. As of 2022, >5 million people worldwide were CRC survivors [5]. The association between obesity and risk of CRC is well established, as risk increases with higher BMI (in kg/m^2^) [[6], [7], [8], [9]]. In a study of 3799 patients with CRC in the United States (1972–2017), 69% had overweight or obesity 6 mo prior to CRC diagnosis [10]. Patients with CRC often have higher body weights due to excessive fat mass (FM), but are at risk of adverse body composition changes, including muscle wasting. Furthermore, inflammation is frequently observed among patients with cancer, and a state of low-grade chronic inflammation is suggested to remain elevated for months to years after cancer treatment in some cancer types [11,12]. These changes can be caused by the cancer itself, the treatment, or insufficient rehabilitation after curative treatment [[13], [14], [15], [16]], ultimately impacting the energy requirements of the patient [17,18].

In the clinic, equations that estimate resting energy expenditure (REE) or basal metabolic rate (BMR) are commonly used to calculate patients’ energy requirements. However, for some cancer types, predictive equations are found to be highly inconsistent in estimating REE, showing that the limits of agreement (LoA) range from ∼40% below and ≤30% above the measured REE [14]. Cancer-related malnutrition is frequently both underestimated and undertreated in clinical practice [19], with potential negative consequences for clinical outcomes, quality of life, patient morbidity, and mortality, as well as prolonging hospital stays and increasing health care expenses [[20], [21], [22]]. Accurate tools for estimating REE in clinical practice are needed to provide adequate nutritional support and avoid underfeeding and overfeeding in patients with cancer and survivors [23,24].

A few studies have measured energy expenditure (EE) in patients with CRC using the ventilated hood connected to a metabolic cart [23,25]. However, the ventilated hood suffers from limitations such as airflow instability and moisture interference, as demonstrated by 10% lower REE estimates compared with the whole-room indirect calorimetry (WRIC) [26]. There is limited research on EE in CRC survivors measured by WRIC [27,28]. The current study is among the first to examine REE in CRC survivors’ after completed cancer treatment. The distinctive aspect of this study is the long-term follow-up assessing that years after curative treatment. We compared estimated REE using predictive equations most commonly used in clinics to REE measured using WRIC. The included equations were the Harris–Benedict, Mifflin–St. Jeor, FAO/WHO/United Nations University (UNU) and Henry equations. Our secondary aim was to determine if body composition-based equations improve agreement and accuracy in predicting REE compared with WRIC.

## Methods

### Study design and population

#### Study design

**FIGURE 1:**
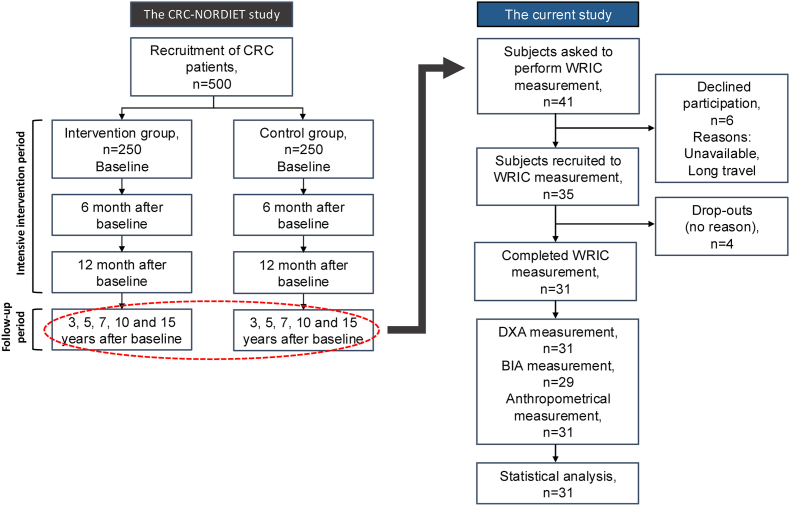
Overview of the current study. The red circle illustrates that the subjects were recruited from the follow-up period of the CRC-NORDIET study. BIA, bioelectrical impedance analysis; CRC, colorectal cancer; DXA, dual-energy X-ray absorptiometry; WRIC, whole-room indirect calorimetry.

In this cross-sectional study, CRC survivors (*n*_total_ = 31, *n*_males_ = 22, *n*_females_ = 9) were recruited from the CRC-NORDIET study [29], to undergo a WRIC measurement to estimate REE (Figure 1).

The WRIC measurement was not part of the standard protocol in the CRC-NORDIET study. To not interfere with the CRC-NORDIET study and protocol, the participant was invited to a separate visit where the REE assessment was completed. The measurements were performed at the Center for Clinical Nutrition at Institution of Basic Medical Sciences, University of Oslo. The CRC-NORDIET study is approved by the Regional Committees for Medical and Health Research Ethics (REC Protocol Approval 2011/836), and by the data protection officials in Oslo University Hospital and Akershus University Hospital. All included subjects in the CRC-NORDIET study provided written informed consents. The CRC-NORDIET study is registered in the National Institutes of Health Clinical Trials (http://www.ClinicalTrials.gov; Identifier: NCT01570010).

#### Subjects and recruitment to WRIC measurement

The included subjects were participants of the CRC-NORDIET study, diagnosed with CRC I–III [30], and treated surgically 2–9 mo before recruitment. The participants of the CRC-NORDIET study were randomly assigned to either an intervention group, receiving a 12-mo intensive dietary intervention and 14 y of maintenance with individual dietary consulting, or a control group, receiving standard clinical care. Both groups received equal advice on physical activity. The nutritional counseling was based on the Norwegian food-based dietary guidelines [31]. Subjects of the current study (*n*_total_ = 31, *n*_intervention_ = 19, *n*_control_ = 12) were recruited from the 3-, 5-, and 10-y follow-up visits of the CRC-NORDIET study. Demographic and clinical data were collected at baseline of the CRC-NORDIET study with a short questionnaire [29]. Baseline characteristics, stage of cancer, treatment regimen and the tumor-node-metastasis classification were obtained from patient’s medical records. Anthropometric measurements, dual-energy X-ray absorptiometry (DXA), and bioelectrical impedance analysis (BIA) were collected at the follow-up visits. The WRIC measurements could not be performed on the exact day of the follow-up visits due to logistical constrains; however, a ± 21-d window of the follow-up visits was considered acceptable, as no major changes in body composition were expected during this short period.

### Whole-room indirect calorimetry

#### Study protocol

**FIGURE 2:**
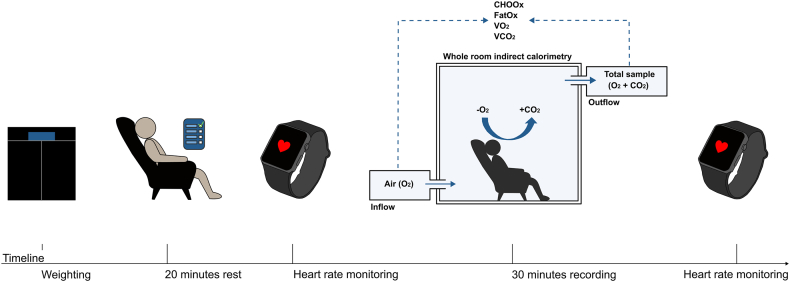
Timeline of the day at whole-room indirect calorimetry measurement of CRC survivors, including preparations and heart rate monitoring. CHOOx, carbohydrate oxidation; CO_2_, carbon dioxide; CRC, colorectal cancer; FatOx, fat oxidation; O_2_, oxygen; VCO_2_, carbon dioxide production; VO_2_, oxygen consumption.

REE (kcal/d) and respiratory quotient [RQ; volume of carbon dioxide production (VCO_2_)/volume of oxygen consumption (VO_2_)] by WRIC were measured in accordance with previously published protocols [26,32]. The calorimetry room contained a toilet, a sink, a mirror, an armchair, and 2 large windows, 1 with a view of the control room, and 1 with an outside view [33]. All subjects were instructed to adhere to 12 h of fasting prior to the WRIC measurement, as well as avoiding caffeine, nicotine, and strenuous physical activity (i.e., heavy weightlifting, long walks >1 h, runs, uphill cycling) the final 24 h before measurement. A total of 60 min were allocated to measure each subject. This included 5 min for weighing (Seca 287) and positioning of a heart rate monitor belt, and 20 min of premeasurement rest to achieve resting heart rate (RHR). The chamber doors were closed 5 min prior to the measurement, which was performed for 30 min. The first and final 5 min of the WRIC measurement were excluded to minimize noise and ensure that only data obtained under steady-state conditions were analyzed. The central 20 min of metabolic data was utilized for extrapolation of all metabolic parameters to 24 h. This meant the mean for each parameter (REE, VO_2_, VCO_2_) was multiplied by 1440 (min in 24 h). For the RQ, only the mean across the 20-min data block was utilized. Subjects’ heart rates were recorded using a heart rate monitor (Polar M400) premeasurement and postmeasurement (Figure 2).

During the measurement, the subjects were instructed to sit as restfully as possible in the armchair. Reading, knitting, and use of mobile phone were allowed to prevent the subjects from falling asleep.

#### Characteristics of the system

The whole-room indirect calorimeter system and protocols for calibration and equilibration performed in the current study have been described previously [26,33,34]. The WRIC has an internal volume of 7500 L. Respiratory gases (VO_2_ and VCO_2_) were measured by the Promethion integrated WRIC system (GA3m2/FG250, Sable Systems International). The data were collected using software Caloscreen 1.3.16 (Sable Systems International), and processed and analyzed using the software ExpeData 1.9.27 (Sable Systems International). REE was calculated using Weir’s equation [35]. Carbohydrate oxidation (CHOOx) and fat oxidation (FatOx) were calculated using Stochiometric equations [36]. The coefficient of variation for REE is 2.68%, as reported in Henriksen et al. [33].

#### Compliance

To determine adherence to the instructions given prior to the WRIC measurement, all subjects completed a compliance sheet consisting of simple yes/no questions, including an open field for the subjects to write down deviations from the protocol, if any. Compliance with premeasurement instructions was monitored to ensure standardized metabolic conditions.

### Anthropometric measurements

All measurements were performed after an overnight fast (≥ 8 h). Height and weight were recorded prior to the DXA scan (see below) using an electronic scale (Seca 285). Subjects wore lightweight clothing with empty pockets and no shoes. Information on height and weight was entered in the DXA software prior to the measurement and used to calculate REE using predictive equations. The subjects were asked about recent weight change in a questionnaire prior to the measurement to detect weight changes of significance (>5 kg) [18,37]. This information was used to ensure metabolic stability (i.e., identify any substantial alterations in health status or underlying conditions that could influence REE).

### Body composition measurements

#### Dual-energy X-ray absorptiometry

The DXA scans were performed according to manufacturer’s protocols [38]. Whole-body DXA scans were conducted using a Lunar iDXA (GE Healthcare Lunar) and software Encore version 18 (GE Healthcare), performed by trained personnel at the CRC-NORDIET follow-up visits. This Lunar iDXA device has shown high precision and valid measurements of body composition compartments in patients with CRC [39,40]. Subjects were scanned in a fasting state lying in a supine position, wearing lightweight clothing. Lean soft tissue (LST) of arms and legs was extracted from the DXA scan to estimate appendicular lean soft-tissue index (ALST index; the sum of LST of arms and legs divided by square height) [41]. For REE prediction (kcal/d), the Encore 18 software applied the Mifflin–St. Jeor equation based on fat-free mass (FFM), referred to as Mifflin–St. Jeor_DXA_ throughout this article (Table 1) [[42], [43], [44], [45]]. The measured FFM by DXA was used in the REE prediction.

**TABLE 1:** Predictive equations utilized to estimate REE and BMR (kcal/d)

Predictive equation	Population	Formula
Harris*–*Benedict [42]	Males	BMR = 66.47 + (13.75 × WT) + (5.003 × HT) – (6.755 × age)
	Females	BMR = 655.1 + (9.563 × WT) + (1.850 × HT) – (4.676 × age)
Mifflin*–*St. Jeor [43]	Males	REE = 9.99 × WT + 6.25 × HT – 4.92 × age + 5
	Females	REE = 9.99 × WT + 6.25 × HT – 4.92 × age – 161
FAO/WHO/UNU [44]	Males	
	30–59 y	BMR = 11.472 × WT + 873.1
	≥60 y	BMR = 11.711 × WT + 587.7
	Females	
	30–59 y	BMR = 8.126 × WT + 845.6
	≥60 y	BMR = 9.082 × WT + 658.5
Henry [45]	Males	
	30–59 y	BMR = 14.2 × WT + 593
	≥60 y	BMR = 13.5 × WT + 514
	Females	
	30–59 y	BMR = 9.74 × WT + 694
	≥60 y	BMR = 10.1 × WT + 569
Predictive equation based on body composition		Formula
Mifflin*–*St. Jeor_DXA_ [43]		REE = 413 + 19.7 × FFM FFM = LST (kg) + BMC (kg)

Abbreviations: BMC, body mineral content; BMR, basal metabolic rate; DXA, dual-energy X-ray absorptiometry; FFM, fat-free mass in kilograms; HT, height in centimeters; LST, lean soft tissue; REE, resting energy expenditure; UNU, United Nations University; WT, weight in kilograms; y, years.

#### Bioelectrical impedance analysis

BIA was performed by the Seca medical Composition Analyzer 515 (Seca) according to previous published protocols [46]. Estimates for REE (kcal/d) prediction were performed using software Seca Analytics 115 (Seca GMBH & Co). The FAO/WHO/UNU equation was incorporated in the Seca Analytic software, used to predict REE [47].

### Predictive equations

Predictive equations based on anthropometric measurements (i.e., height, weight) and body composition (i.e., FFM) were utilized to estimate REE (kcal/d) of all subjects (Table 1). The FAO/WHO/UNU equation was used twice; 1 with weight obtained from the electronic scale (Seca 285), referred to as the conventional FAO/WHO/UNU equation, and 1 with weight obtained by the software Seca Analytics 115 (Seca GMBH & Co), referred to as the FAO/WHO/UNU_BIA_ equation.

### Sample size estimation

Using *α* = 0.05, *β* = 0.20 (80% power), an expected mean difference of 12%, and an SD of 6%, we calculated that ∼24 subjects were required to estimate the LoA with adequate precision [48]. The anticipated LoA was set to ± 30%. We recruited 31 subjects to allow for ≤25% loss due to protocol deviations. The parameters used for sample size estimation were based on an internal pilot dataset, and pragmatic considerations with respect to the trial schedule and time constraints of this subproject and the participants.

### Data processing and statistical analysis

Data distribution was evaluated through visual inspection of histograms, Q-Q plots, detrended Q-Q plots, and box plots. Descriptive statistics are presented as mean (SD) and median (min–max) for continuous variables to give a detailed view of the distribution. Categorical variables are presented as counts (%). Paired sample t-test was used to determine the difference between measured REE_WRIC_ and predicted REE. Spearman’s rank correlation coefficients (*r*) were calculated for REE_WRIC_ and REE predicted by equations to assess the relationship between measured and predicted REE [49]. To examine the practical importance between the 2 methods used, effect sizes are expressed as Cohen’s *d* where *r* = 0.20, 0.50, and 0.80 indicate small, medium, and large, respectively [50].

To assess the individual-level agreement between REE_WRIC_ measured and predicted REE, modified Bland–Altman analysis [51] was performed and graphically presented as Bland–Altman plots. The LoA was defined as mean ratio ± 1.96 SD, with REE_WRIC_ treated as a criterion method. To examine systematic bias (intercepts) and proportional bias (slope), Passing–Bablok linear regression was used. An intercept confidence interval (CI) including 0 indicated no systematic bias, whereas a slope CI including 1 indicated no proportional bias [52]. Lin’s concordance correlation coefficient (CCC) was estimated to assess the precision and accuracy of predicted REE compared with REE_WRIC_. Values > 0.99 indicated strong agreement, and values < 0.90 indicated poor concordance between the predicted and measured REE [53]. The mean absolute percentage error (MAPE), based on mean ratio, was calculated to assess the statistical accuracy of the predicted REE from each equation compared with the measured REE_WRIC_. Accuracy was classified as high with values <10%, and poor with values >51% according to Lewis’s interpretation of MAPE [54]. The MAPE, which is based on mean ratio, does not reflect directional bias and may yield a more favorable result than when the mean difference is used. For supplementary analysis, Passing–Bablok plots were generated to assess the relationship between equation-derived REE and REE_WRIC_.

To assess which of the commonly used equations are the most preferable in clinical use, we evaluated the performance of the equations at group-level perspective. Specifically, by calculating the accuracy of predicted REE by determining the percentage of subjects with predicted REE values within ± 10% of REE_WRIC_, the cut-off for what is considered to be clinically relevant [55]. Within this range, REE predictions were considered accurate, whereas those falling above and below were deemed overestimations or underestimations, respectively.

Sensitivity analyses were conducted to assess the potential effects of the intervention and follow-up in both measured and predicted REE. Because sex is an important component of REE [17,18,56], we also conducted analyses stratified by sex. The differences in measured REE_WRIC_ between sexes, treatment group, and follow-up year were tested with the Wilcoxon signed-rank test. The agreement between predicted and measured REE at the individual level was assessed using modified Bland–Altman plots, stratified by sex, treatment group, and follow-up year. These plots were based on the mean ratio, with WRIC treated as the criterion method.

To facilitate comparison with previous published studies, Bland–Altman analysis based on the mean difference between the methods and mean bias-based LoA was calculated [51]. Linear regression analyses [49] were conducted in sensitivity analysis to assess any proportional bias, where the difference between the methods was considered the dependent variable, whereas the mean of the 2 methods served as the independent variable. To elucidate possible impact from lack of adherence to the study protocol for 9 subjects, sensitivity analyses among fully compliant subjects were performed (*n* = 22). Statistical analyses were prepared using Microsoft Excel 2016 and performed in IBM SPSS Statistics version 29.0.0.0, for Windows/Mac, and R version 4.5.1 (R Foundation for Statistical Computing). Data processing and figure preparation were performed using the tidyverse packages (dplyr, ggplot2, and tidyr) and the mcr package. The statistical significance level was set to *P* < 0.05.

## Results

From the CRC-NORDIET study, 41 subjects were invited to participate in this study. Of these, 35 agreed to undergo a WRIC measurement. Four subjects dropped out for no stated reason, resulting in a final study sample of 31 subjects (Figure 1).

### Characteristics

The clinical, anthropometric, and demographic characteristics of the included subjects are listed in Table 2. The median (min–max) of the interval between the WRIC measurement and the follow-up visit date was 7 (4–17) d. Of the subjects, 29% were females and the mean (SD) age was 69 (6.90) y. Mean body weight of the total study population was 89.8 (18.1) kg, and according to the BMI groups defined by the WHO, the majority were classified having overweight (48%) or obesity (36%) [57]. For body composition, mean FM was 31.9 (8.34) kg and mean FFM was 57.2 (11.9) kg. Variables for body composition and anthropometric measurements by sex are presented separately in Table 3. Males had significantly higher FM, visceral adipose tissue, and lean mass (FFM, LST, ALST index) than females (*P* < 0.05). Mean RHR of the included subjects was 63.3 (5.34) beats per minute (bpm) before, and 62.9 (6.57) bpm post WRIC measurement.

**TABLE 2:** Characteristics of the included subjects (*n* = 31)^1^

Demographic variables	*n* (%)	Median (min–max)
Age^2^ (y)	69 (6.90)	69 (53–78)
Sex		
Males	22 (71)	—
Females	9 (29)	—
Clinical variables		
Tumor localization^3^		
C18 colon	19 (66)	—
C19 rectosigmoid	1 (3)	—
C20 recti	9 (31)	—
TNM stage		
I	10 (32)	—
II	12 (39)	—
III	9 (29)	—
Additional treatment		
Neoadjuvant chemotherapy	2 (7)	—
Adjuvant chemotherapy	9 (29)	—
Years since surgery		
3 y	17 (55)	—
5 y	8 (26)	—
10 y	6 (19)	—

Abbreviation: TNM, tumor-node-metastasis staging system.

1 I–III describes the extent of the malignant disease.

2 Values are presented as mean (SD).

3 *n* = 29; 2 people missing tumor localization data.

**TABLE 3:** Body composition and anthropometric measurements in males and females (*n* = 31)

Body composition^1^	Total (*n* = 31)	Males (*n* = 22)	Females (*n* = 9)
Weight (kg)	88.0 (56.3–130)	96.6 (80.8–130)	69.5 (56.2–88.0)
Height (cm)	177 (155–194)	181 (168–194)	168.9 (155–173)
WC (cm)	102 (77.3–128)	107 (86.0–128)	82.0 (77.3–93.0)
BMI (kg/m^2^)	28.0 (20.5–37.8)	29.3 (22.9–37.8)	25.2 (20.5–30.5)
FM/FFM^2^	0.56 (0.29–0.91)	0.55 (0.29–0.76)	0.65 (0.42–0.91)
BMI categories^3^ (kg/m^2^)			
18.5–24.9	5 (16)	1 (4.50)	4 (44.4)
25–29.9	15 (48)	11 (50.0)	4 (44.4)
>30	11 (36)	10 (45.5)	1 (11.2)
FM^2^			
FM (kg)	30.8 (17.1–55.4)	33.5 (20.2–55.4)	26.0 (17.1–41.5)
VAT (g)	2133 (68.4–4427)	2356 (793–4427)	570 (68.4–1652)
SAT (g)	1597 (696–3565)	1716 (981–3565)	1523 (696–2185)
Muscle-related compartments^2^			
FFM (kg)	60.6 (34.6–76.1)	64.2 (48.6–76.1)	41.0 (34.6–48.2)
LST (kg)	57.4 (32.7–71.9)	60.9 (46.0–71.9)	39.1 (32.7–45.1)
ALST index (kg/m^2^)	7.95 (5.67–10.3)	(0.88 (0.68–1.03)	0.63 (0.57–0.74)

Abbreviations: ALST, appendicular lean soft tissue; DXA, dual-energy X-ray absorptiometry; FFM, fat-free mass; FM, fat mass; LST, lean soft tissue; SAT, subcutaneous adipose tissue; VAT, visceral adipose tissue; WC, waist circumference.

1 All values are presented in median (min–max).

2 The variables are measured by and calculated from body composition values measured by DXA.

3 Values are presented as counts (percentage).

Among the subjects, 55% had undergone curative surgery 3 y ago, whereas 26% and 19% had undergone curative surgery 5 and 10 y ago, respectively. Eleven subjects (35%) of the total population had previously received additional treatment, with 29% received adjuvant chemotherapy and 7% received neoadjuvant chemotherapy.

### Metabolic measurements of WRIC

The parameters measured by WRIC and the predicted REE by equations are presented in Table 4. Mean (SD) REE_WRIC_ was 1710 (353) kcal/d. For substrate oxidation, mean RQ (SD) was 0.79 (0.05), CHOOx was 107 (73.9) g/d and FatOx was 95.1 (40.8) g/d. Mean VO_2_ was 355 (73.5), whereas mean VCO_2_ was 280 (59.8) L/d. The measured REE_WRIC_ and predicted REE by equations are presented by sex in Supplemental Table 1. Males had significantly higher REE, FatOx, VO_2_, and VCO_2_ compared with females (*P* < 0.05).

**TABLE 4:** Metabolic variables measured with WRIC and predicted REE by included equations (*n* = 31)

WRIC variable	Mean (SD)	Median (min–max)			
REE (kcal/d)	1710 (353)	1711 (1168–2213)	—	—	—
RQ (VO_2_/VCO_2_)	0.79 (0.05)	0.79 (0.70–0.92)	—	—	—
CHOOx (g/d)	107 (73.9)	98.2 (0.38–317)	—	—	—
FatOx (g/d)	95.1 (40.8)	97.1 (21.7–197)	—	—	—
VO_2_ (L/d)	355 (73.5)	353 (240–454)	—	—	—
VCO_2_ (L/d)	280 (59.8)	305 (186–396)	—	—	—

Equation	REE (kcal/d), mean (SD)	REE (kcal/d), median (min–max)	Diff^1^ (kcal/d), mean (95% CI)	*P*^2^	Cohen’s *d*

Harris–Benedict	1697 (309)	1749 (1188–2275)	–12.3 (–76.2, 51.6)	0.710	0.07
Mifflin–St. Jeor	1610 (289)	1694 (1062–2083)	–99.3 (–167, –32.2)	0.007	0.52
FAO/WHO/UNU	1628 (258)	1619 (1170–2106)	–81.5 (–153, –10.5)	0.032	0.40
Henry	1695 (309)	1703 (1166–2264)	–14.8 (–80.1, 50.4)	0.660	0.08
Mifflin–St. Jeor_DXA_	1539 (234)	1608 (1094–1912)	–170 (–241, –99.9)	<0.001	0.85
FAO/WHO/UNU_BIA_^3^	1693 (298)	1764 (1204–2220)	0.90 (–65.6, 67.4)	0.979	–0.01

Abbreviations: BIA, bioelectrical impedance analysis; CHOOx, carbohydrate oxidation; CI, confidence interval; DXA, dual-energy X-ray absorptiometry; FatOx, fat oxidation; L/d, liter per day; REE, resting energy expenditure; RQ, respiratory quotient; UNU, United Nations University; VCO_2_, carbon dioxide production; VO_2_, oxygen consumption; WRIC, whole-room indirect calorimetry.

1 Mean difference from REE_WRIC_.

2 Paired-sampled *t*-test was used to compare REE_WRIC_ and REE predicted by each equation.

3 *n* = 29.

Predicted REE from the Mifflin–St. Jeor, FAO/WHO/UNU, and Mifflin–St. Jeor DXA equations was significantly lower than measured REE_WRIC_ (*P* < 0.05), with medium-to-large effect sizes (*d* = 0.40–0.85). No significant differences were observed between WRIC and the Harris–Benedict, Henry or FAO/WHO/UNU_BIA_ equations (*P* > 0.660) with small effect sizes (*d* = –0.01–0.08). No significant differences were found between the intervention and control groups in measured REE_WRIC_ and predicted REE by equations (Supplemental Table 2).

### Agreement between measured and predicted REE values

**FIGURE 3:**
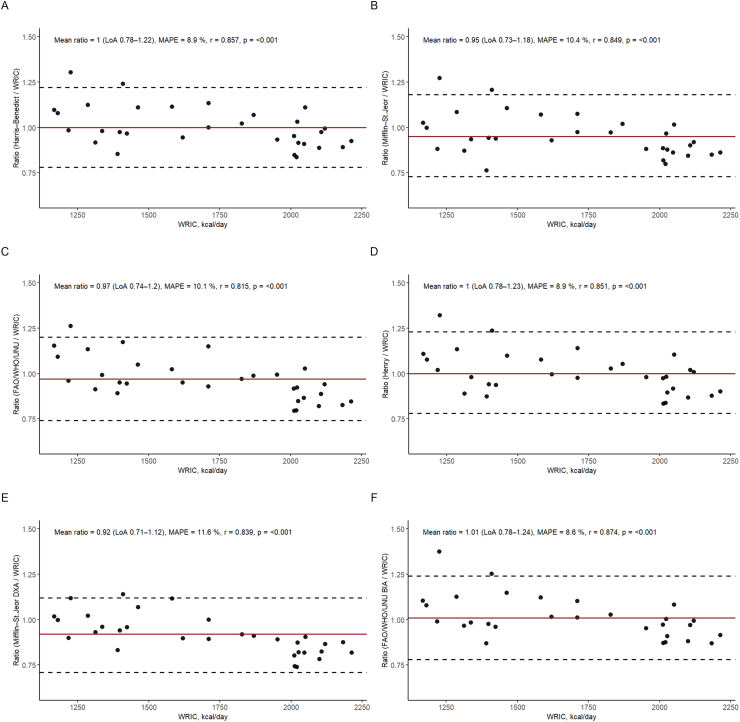
(A–F) Bland –Altman plots showing the mean ratio between 2 methods for estimating REE against the criterion (measured REE). The REE was estimated in CRC survivors (*n* = 31; FAO/WHO/UNU_BIA_, *n* = 29). (A) Harris–Benedict vs. WRIC. (B) Mifflin–St. Jeor vs. WRIC. (C) FAO/WHO/UNU vs. WRIC. (D) Henry vs. WRIC. (E) Mifflin–St. Jeor_DXA_ vs. WRIC. (F) FAO/WHO/UNU_BIA_ vs. WRIC. BIA, bioelectrical impedance analysis; CRC, colorectal cancer; DXA, dual-energy X-ray absorptiometry; LoA, limits of agreement; MAPE, mean absolute percentage error; *p*, *P* value from correlation analysis presenting the significance of correlation; *r*, correlation coefficient form Spearman correlation analysis showing relation between WRIC and the other methods; REE, resting energy expenditure; UNU, United Nations University; WRIC, whole-room indirect calorimetry.

The agreement between estimated and measured REE values, including LoA and MAPE, is illustrated in Bland–Altman plots (Figure 3A–F).

Mean ratios and LoA indicated that most equations underestimated REE to a varying degree. Overall, Mifflin–St. Jeor_DXA_ underestimated REE the most by 8% (Figure 3E). The ratio-based LoAs ranged from –22% to 22% for Harris–Benedict, –27% to 18% for Mifflin–St. Jeor, –26% to 12% FAO/WHO/UNU, –22% to 23% for Henry, –29% to 12% for Mifflin–St. Jeor_DXA_, and 22% to 24% for FAO/WHO/UNU_BIA_. The MAPE was highest for Mifflin–St. Jeor_DXA_ and lowest for Harris–Benedict, Henry, and FAO/WHO/UNU_BIA_ equations (Figure 3A, D, E, and F).

Bland–Altman plots stratified by sex, treatment group, and follow-up time are shown in the supplementary materials (Supplemental Figures 1A–F, 2A–F, and 3A–F). REE across follow-up periods is presented in Supplemental Table 3.

The conventional Bland–Altman analysis using absolute values is presented in the supplementary materials (Supplemental Table 4). For the mean bias-based LoA of predicted REE by equations, we found that LoA ranged from –33% to 21%, compared with REE_WRIC_. The LoA of predicted REE ranged from –22% to 20% for the Harris–Benedict, and Henry equations and from –21% to 21% for the FAO/WHO/UNU_BIA_ equation.

### Passing–Bablok linear regression and Lin’s CCC

The results from Passing–Bablok linear regression and Lin’s CCC are presented in Table 5. Mifflin–St. Jeor_DXA_ showed the poorest agreement from REE_WRIC_, with both systematic and proportional bias indicating underestimation of REE at higher values. Proportional bias was also found for FAO/WHO/UNU, whereas no bias was found for Mifflin–St. Jeor, Henry, Harris–Benedict or FAO/WHO/UNU_BIA_ equations. Agreement analyses using Lin’s CCC supported these findings. The agreement with REE_WRIC_ was lowest for Mifflin–St. Jeor_DXA_ and FAO/WHO/UNU intermediate for Mifflin–St Jeor, and highest for Harris–Benedict, Henry, and FAO/WHO/UNU_BIA_. Among the predictive equations, Harris–Benedict, Henry, and FAO/WHO/UNU_BIA_ showed the best overall agreement with REE_WRIC_, with slopes closest to 1 and the highest CCC. The relationship between REE predicted by equations and measured REE_WRIC_ is illustrated in Passing–Bablok regression plots provided in the supplementary materials (Supplemental Figure 4A–F).

**TABLE 5:** Passing–Bablok linear regression and Lin’s CCC of predictive equations against REE (kcal/d) measured by WRIC^1^

Equation	Intercept (95% CI)	Slope (95% CI)	Lin’s CCC (95% CI)
Harris–Benedict	211 (–177, 473)	0.85 (0.69, 1.10)	0.85 (0.72, 0.92)
Mifflin–St. Jeor	220 (–109, 467)	0.79 (0.65, 1.02)	0.79 (0.63, 0.88)
FAO/WHO/UNU	360 (–28.9, 627)	0.73 (0.56, 0.97)	0.76 (0.59, 0.86)
Henry	182 (–277, 479)	0.88 (0.70, 1.14)	0.84 (0.71, 0.92)
Mifflin–St. Jeor_DXA_	405 (199, 641)	0.66 (0.52, 0.79)	0.67 (0.49, 0.79)
FAO/WHO/UNU_BIA_	283 (–69.8, 552)	0.84 (0.67, 1.04)	0.85 (0.71, 0.92)

Abbreviations: BIA, bioelectrical impedance analysis; CI, confidence interval; DXA, dual-energy X-ray absorptiometry; Lin’s CCC, Lin’s concordance correlation coefficient; REE; resting energy expenditure; UNU, United Nations University; WRIC, whole-room indirect calorimetry.

1 Intercept (systematic bias) and slope (proportional bias) between the measured REE and the predicted REE was estimated using Passing–Bablok linear regression. Precision and accuracy were estimated using Lin’s CCC.

### Accuracy of the predicted REE

**FIGURE 4:**
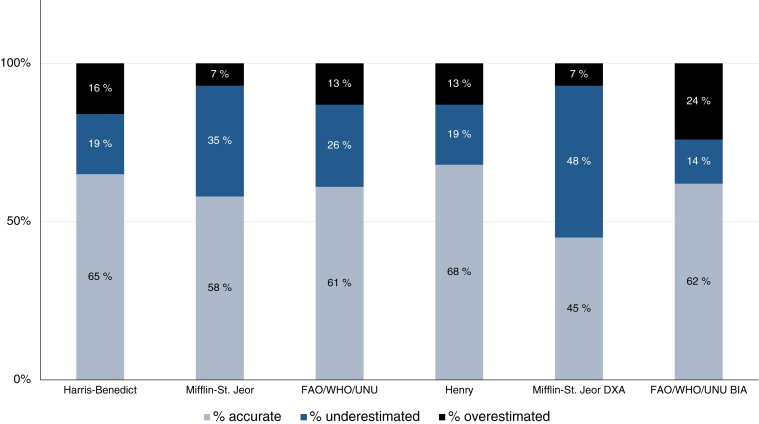
The accuracy of the predicted REE in CRC survivors (*n* = 31; FAO/WHO/UNU_BIA_, *n* = 29) is illustrated as the percentage of REE values that fell within and outside ± 10% of REE_WRIC_. Predicted values within these accuracy limits were considered accurate, and values above and below these accuracy limits were considered overestimation and underestimations, respectively. BIA, bioelectrical impedance analysis; CRC, colorectal cancer; DXA, dual-energy X-ray absorptiometry; REE, resting energy expenditure; UNU, United Nations University; WRIC, whole-room indirect calorimetry.

The accuracy of the predicted REE by the equations, characterized by the percentage of predicted REE values within ± 10% of REE_WRIC_, is presented in Figure 4.

All equations tended to underestimate REE except for the FAO/WHO/UNU_BIA_ equation. The lowest accuracy was observed by the Mifflin–St. Jeor_DXA_ equation. The Harris–Benedict, Henry and FAO/WHO/UNU_BIA_ equations showed the highest accuracy.

### Compliance to protocol

Results from sensitivity analysis performed after excluding subjects that did not adhere to the study protocol (fasting, *n* = 2; nicotine, *n* = 2; caffeine, *n* = 2; strenuous activity, *n* = 2; nicotine and strenuous activity, *n* = 1) are shown in Supplemental Figure 5A–F. The overall underestimation of REE by prediction equations compared with REE_WRIC_ was reduced when only fully compliant subjects were included in the analysis (*n* = 22). For all methods, the trends and patterns were consistent with the primary analyses (*n* = 31).

## Discussion

In this cross-sectional study, REE estimated from commonly used predictive equations was compared with REE_WRIC_. Our findings indicate that although the equations performed reasonably well at the group level, they had poor individual-level agreement. A systematic difference that increased with higher REE was observed among several equations. Overall, 19%–48% of the predicted REE values fell outside the ± 10% accuracy limit of measured REE_WRIC_ and were thus considered underestimations.

Evidence on REE in CRC survivors’ years after curative surgery is limited. However, during the preparation of this manuscript, Pagano et al. [27] published a similar study where REE estimated by predictive equations were compared with REE_WRIC_ among CRC survivors (*n* = 20) assessed within 3 y of postcancer treatment. Similarly to our results, they observed that equations tended to underestimate REE in CRC survivors. Among these, the Harris–Benedict equation demonstrated the best individual-level agreement. For accuracy, Pagano et al. [27] reported results consistent with ours for the Harris–Benedict (65% accurate) and Henry (60% accurate), whereas lower accuracy and more underestimations were observed for the FAO/WHO/UNU (i.e., Schofield weight), Mifflin–St. Jeor, and Mifflin–St. Jeor with FFM. These findings support that the Harris–Benedict and Henry equations were the most accurate on the group level, whereas Harris–Benedict also performed better at the individual level. The discrepancy between the studies may be explained by the differences in time since treatment. Earlier inclusion may entail incomplete recovery and post cancer treatment inflammation leading to elevated REE and reduced accuracy of the equations [58]. Our findings further support previous studies reporting inaccurate REE predictions from equations in patients with various cancer types and survivors, where equations were compared with REE from ventilated hoods [28,55].

With respect to agreement, accuracy, and proportional bias, the Harris–Benedict, Henry and FAO/WHO/UNU_BIA_ equations performed better than the other equations, but substantial individual variability was present, making them less reliable in clinical care. Notably, 19% of the REE estimates predicted by Harris–Benedict and Henry equations underestimated REE by >10%. The source of this high variability is not clear; however, none of the included equations were developed with the intention to predict REE in patients with complex disease conditions, which is known to increase REE [59]. The FAO/WHO/UNU_BIA_ was the only equation that tended to overestimate predicted REE, which may be associated with an elevated mean extracellular water/total body water (TBW) ratio (> 0.40) despite normal TBW [[60], [61], [62], [63]].

It is important to consider that the majority of the subjects in this study had overweight or obesity. Most existing predictive equations do not account for body composition beyond weight and height, and excessive adipose tissue can reduce their accuracy by masking potential muscle loss [64,65]. Therefore, we included Mifflin–St. Jeor_DXA_, which accounted for FFM, and which we expected to align more closely with REE_WRIC_. DXA scans are particularly relevant since FFM and FM can vary significantly among patients with cancer, independent of sex and body size [23,55]. FFM is considered a metabolically active component of body composition, and differences among the subjects are relevant to affect REE [[66], [67], [68]]. However, Mifflin–St. Jeor_DXA_ largely underestimated REE with 48% of values falling outside the accuracy limits. Moreover, Mifflin–St. Jeor_DXA_ showed proportional bias indicating greater underestimations of REE at higher REE values. This likely reflects that subjects with overweight and obesity—who typically have higher REE and a greater portion of FM—were overrepresented at higher REE values [69,70].

Our results support previous findings that predictive equations considering body composition measures are inconsistent with REE measured using indirect calorimetry (IC) [55]. Notably, the Mifflin–St. Jeor_DXA_ equation has been developed based on skinfold thickness and circumference, which are known to be far less accurate than body composition measured by DXA [43]. This suggests that the limitation lies in the equation, rather than the inclusion of body composition when predicting REE. Mifflin–St. Jeor_DXA_ also excludes data on sex, another significant component of REE [17].

Another point to consider is that most equations (Harris–Benedict, Henry, FAO/WHO/UNU) are intended to estimate BMR [42,44,45], which requires stricter conditions (and consequently lower EE) than what we implemented in the present study [17,71], potentially creating a larger gap between measured and predicted REE for these equations. Paradoxically, the equations that estimate BMR showed the most accurate results with fewer underestimations compared with the other equations evaluated. However, reliance on older closed-circuit IC methods may lead the Harris–Benedict and FAO/WHO/UNU equations to overestimate BMR [45,72], whereas adherence of the Henry equation to strict BMR protocols is not fully described.

The impact of the cancer burden and treatment effects in CRC survivors 3–10 y after curative surgery is unclear. Survivors may have experienced large alterations in body composition during treatment and rehabilitation, from which they have not fully recovered [73]. Chronic inflammation is suggested to persist in the body years after curative cancer treatment, potentially providing sustained higher energy requirements [12,74]. Considering age-related muscle mass decline, adapting to a healthy body weight after surgery could be challenging, even years after curative surgery. This may result in sustained low REE, which should be considered for this population, pending further research [75,76].

It is well known that patients with cancer in active disease commonly experience a metabolic shift toward altered glucose oxidation and excessive mobilization of fat [77,78]. Because REE measurements using WRIC provides data on substrate oxidation, a brief discussion is warranted. The mean RQ of 0.79 indicated primarily FatOx among the subjects. This is partly in contrast to Ohmae et al. [79], who reported a mean (min–max) RQ of 0.88 (0.85–0.95) in patients with head- and neck cancer, suggesting a mix of both fat and carbohydrate utilization. Note that the metabolic cart is known to underestimate RQ compared with WRIC [26], and use of different methods, precludes comparison. The clinical relevance of these findings is yet to be established.

A strength of this study is the unique and well-characterized population consisting of CRC survivors years after curative treatment. To our knowledge, this is one of the first studies presenting REE data in this population, which is of significant clinical relevance for CRC survivors. Furthermore, we have used state-of-the-art equipment for the measurement of REE, and the specific WRIC chamber used in this study has recently been validated [33].

A considerable limitation is the small sample size and uneven sex distribution in the final study sample. The resulting variability is reflected in wide CIs in tests for proportional and systematic bias, and should be considered when cautiously interpreting our results. However, we note that our sample size is comparable to similar studies [27,28], with comparable results, strengthening the evidence base for this hard-to-reach population. Another limitation is that some subjects underwent a 12-mo intensive dietary intervention and subsequent maintenance phase, potentially affecting their EE through effects on body composition. However, a separate study on the CRC-NORDIET population showed no significant difference in FFM between the intervention and the control group after 12 mo [80]. Furthermore, sensitivity analyses did not indicate differences based on intervention allocation in the parent study. Finally, our findings cannot be generalized to individuals with cancer, as survivors who have completed curative treatment have different burden of tumor-derived factors, cancer treatment and subsequent effects on body composition [59]. Patients with CRC often have overweight or obesity, which further precludes generalization to other patients with cancer, although previous studies report underestimations of energy requirements in several cancer types [81]. Future longitudinal studies measuring REE in newly diagnosed patients with CRC and after a period of follow-up may give more accurate answers.

In conclusion, predictive equations commonly used in the clinic underestimate REE in CRC survivors 3–10 y after curative treatment, and the poor individual agreement limits the clinical applicability to the equations. This highlights the urgent need for better tools to estimate energy requirements in clinical care to ensure adequate nutritional recommendations in cancer survivors. On the basis of the performance of Mifflin–St. Jeor_DXA_, future studies are needed to develop predictive equations based on actual body composition compartments by DXA for increased accuracy in REE estimation based on FFM.

## Author contributions

The authors’ responsibilities were as follows – TO, DTA, DMK, RB: designed the study; RRE: conducted the study; RR: customized the macro files used to estimate metabolic rates; RB, DTA, ÅK, RR, TO: provided essential materials to perform the study; RRE, TO, ÅK: performed statistical analysis; RRE, TO: drafted the manuscript and had primarily responsibility for final content; and all authors: read, revised, and approved the final manuscript.

## Data availability

Data described in the manuscript, code book, and analytic code will not be made available because (highly sensitive data).

## Declaration of generative AI in and AI-assisted technologies in the writing process

During the preparation of this work, the authors used “Wordtune Editor,” “QuillBot,” and “ChatGPT” in order to improve language. After using these tools, the authors reviewed and edited the content as needed and takes full responsibility for the content of the publication.

## Funding

This project has received support from Research Council of Norway, The Throne Holst Nutrition Research Foundation of research and the University of Oslo.

## Conflict of interest

The authors report no conflicts of interest. RR is the owner of D&S Consulting, which provides services related to calorimetry rooms. RR declares no conflict of interest in that no financial gain was achieved from the data produced for this manuscript.
