# Supplementary material for: Predictive equations commonly used in the clinic underestimate resting energy expenditure compared with whole-room indirect calorimetry in colorectal cancer survivors
Source: Am J Clin Nutr. 2026 Jan 27;123(3):101209. doi: 10.1016/j.ajcnut.2026.101209 (PMC12975359; doi:10.1016/j.ajcnut.2026.101209)
Supplement: Multimedia component 1 [file mmc1.pdf]

Title: Predictive equations commonly used in clinics underestimate resting energy expenditure compared with whole-room indirect calorimetry in colorectal cancer survivors
First Author: Rakel Raknes Eklo

Supplementary Materials

Supplementary Table 1: Resting energy expenditure and substrate oxidation measured by WRIC and REE measured by predictive equations in males and females[1,2]

**WRIC variable**	**Males (n = 22)**	**Females (n = 9)**	***P***[3]
**REE**, kcal/d	1876 ± 273 (1225–2213)	1302 ± 95.7 (1168–1423)	< 0.001
**RQ**, VO_2/VCO_2	0.79 ± 0.05 (0.69–0.92)	0.78 ± 0.04 (0.71–0.84)	NS
**CHOOx**, g/d	122.8 ± 78.9 (0.40–317)	72.9 ± 35.9 (12.1–124)	NS
**FatOx**, g/d	106 ± 41.5 (21.7–197)	69.0 ± 25.2 (31.3–134)	< 0.05
**VO_2**, L/d	390 ± 57.1 (253–454)	271 ± 20.9 (240–295)	< 0.001
**VCO_2**, L/d	304 ± 46.5 (208–396)	212 ± 16.0 (186–243)	< 0.001
**Equation**[4]			
**Harris Benedict**	1863 ± 186 (1718–1984)	1292 ± 88.9 (1203–1360)	< 0.001
**Mifflin-St. Jeor**	1771 ± 144 (1673–1888)	1216 ± 117 (1143–1316)	< 0.001
**FAO/WHO/UNU**	1762 ± 163 (1612–1869)	1299 ± 87.9 (1239–1344)	< 0.001
**Henry**	1861 ± 185 (1694–1965)	1289 ± 82.4 (1242–1313)	< 0.001
**Mifflin-St. Jeor$_{DXA}$**	1664 ± 139 (1573–1758)	1235 ± 88.9 (1176–1314)	< 0.001
**FAO/WHO/UNU$_{BIA}$**[5]	1869 ± 155 (1761–1972)	1304 ± 78.7 (1267–1363)	< 0.001

[1]BIA, bioelectrical impedance analysis; DXA, dual-energy x-ray absorptiometry; CHOOx, carbohydrate oxidation; FatOx, fat oxidation; NS, non-significant; REE, resting energy expenditure; RQ, respiratory quotient; VCO_2, carbon-dioxide production; VO_2, oxygen consumption; WRIC, whole-room indirect calorimetry.

[2]Values are presented as mean ± SD (min–max).

[3]Wilcoxon rank-sum test was used to compare REE_{WRIC}, REE by each equation and substrate oxidation between males and females.

[4]All values are presented in kcal/d.

[5]Males, n = 20; Females, n = 9.

Supplementary Table 2: Measured and predicted REE (kcal/d) in the intervention group and the control group[1,2]

	Intervention	Control	Diff[2]	*P*
**WRIC**	1761 ± 342 (1168–2213)	1628 ± 370 (1168–2213)	133.1	NS
**Harris-Benedict**	1695 ± 306 (1188–2275)	1701 ± 328 (1199–2107)	-5.4	NS
**Mifflin-St. Jeor**	1611 ± 284 (1062–2083)	1609 ± 311 (1072–1952)	1.6	NS
**FAO/WHO/UNU**	1632 ± 254 (1196–2106)	1622 ± 274 (1170–1994)	9.5	NS
**Henry**	1693 ± 307 (1166–2264)	1697 ± 325 (1242–2148)	-4.3	NS
**Mifflin-St. Jeor$_{DXA}$**	1538 ± 227 (1157–1912)	1541 ± 255 (1094–1833)	-2.2	NS
**FAO/WHO/UNU$_{BIA}$**	1691 ± 294 (1209–2220)	1697 ± 31.6 (1204–2105)	-6.5	NS

[1]BIA, bioelectrical impedance analysis; DXA, dual-energy x-ray absorptiometry; NS, non-significant; REE, resting energy expenditure; WRIC, whole-room indirect calorimetry.

[2]Values are presented as mean ± SD (min–max).

[3]Wilcoxon rank-sum test was used to compare REE_{WRIC} and REE by equations between the intervention group and the control group.

Supplementary Table 3: Resting energy expenditure (kcal/d) across follow-up periods[1]

**Equation[2]**	**3-year, Median (min–max)**	**5-year, Diff (95 % CI)[3]**	**10-year, Diff (95 % CI)[3]**
**REE_{WRIC}**	1711 (1168–2213)	-10.3 (-331, 311)	-16.7 (372, 339)
**Harris-Benedict**	1708 (1188–2275)	71.90 (-208, 352)	22.48 (-287, 332)
**Mifflin-St. Jeor**	1666 (1062–2083)	99.1 (-161, 359)	44.1 (-244, 332)
**FAO/WHO/UNU**	1598 (1170–2106)	81.4 (-151, 313)	15.9 (-241, 273)
**Henry**	1679 (1166 –2264)	81.7 (-197, 361)	13.9 (-295, 323)
**Mifflin-St. $Jeor_{DXA}$**	1526 (1094–1912)	83.3 (-127, 294)	29.4 (-204, 262)
**$FAO/WHO/UNU_{BIA}$[4]**	1753 (1204–2220)	78.9 (-204, 362)	-14.8 (-336, 306)

[1]BIA, bioelectrical impedance analysis; CI, confidence interval; DXA, dual-energy x-ray absorptiometry; REE, resting energy expenditure; WRIC, whole-room indirect calorimetry.

[2]Values are presented in kcal/d.

[3]Values are presented in mean difference from 3-year follow-up as reference along with difference in REE between the follow-up timepoints and 95 % coefficient.

Supplementary Table 4: Bland-Altman limit analysis for comparison between predicted and estimated REE (kcal/d) in CRC survivors (n = 31)[1]

**Comparison**[2]	**Bias**[3], **Mean (min–max)**	**Limits of agreement**		**Proportional bias ($p < 0.05$)**
30– min WRIC measurement **vs.** Harris-Benedict equation	-12.3 (-334–370)	-368	343	NS
30– min WRIC measurement **vs.** Mifflin-St. Jeor equation	-99.3 (-407–332)	-473	275	0.05
30– min WRIC measurement **vs.** FAO/WHO/UNU equation	-81.5 (-414–320)	-477	314	< 0.05
30– min WRIC measurement **vs.** Henry equation	-14.8 (-334–392)	-378	348	NS
30– min WRIC measurement **vs.** Mifflin-St. Jeor DXA equation	-170 (-526–198)	-562	221	< 0.001
30– min WRIC measurement **vs.** Mifflin-St. Jeor BIA equation[4]	1.03 (-287–458)	-357	359	NS

[1]BIA, bioelectrical impedance analysis; DXA, dual-energy x-ray absorptiometry; CRC, colorectal; NS, non-significant; REE, resting energy expenditure; WRIC, whole-room indirect calorimetry.

[2]The data from 30-minute WRIC measurement is extrapolated to 24-hours.

[3]Mean difference between the REE_{WRIC} and REE estimated by equations.

[4]n = 29.

Supplementary Figure 1A–F

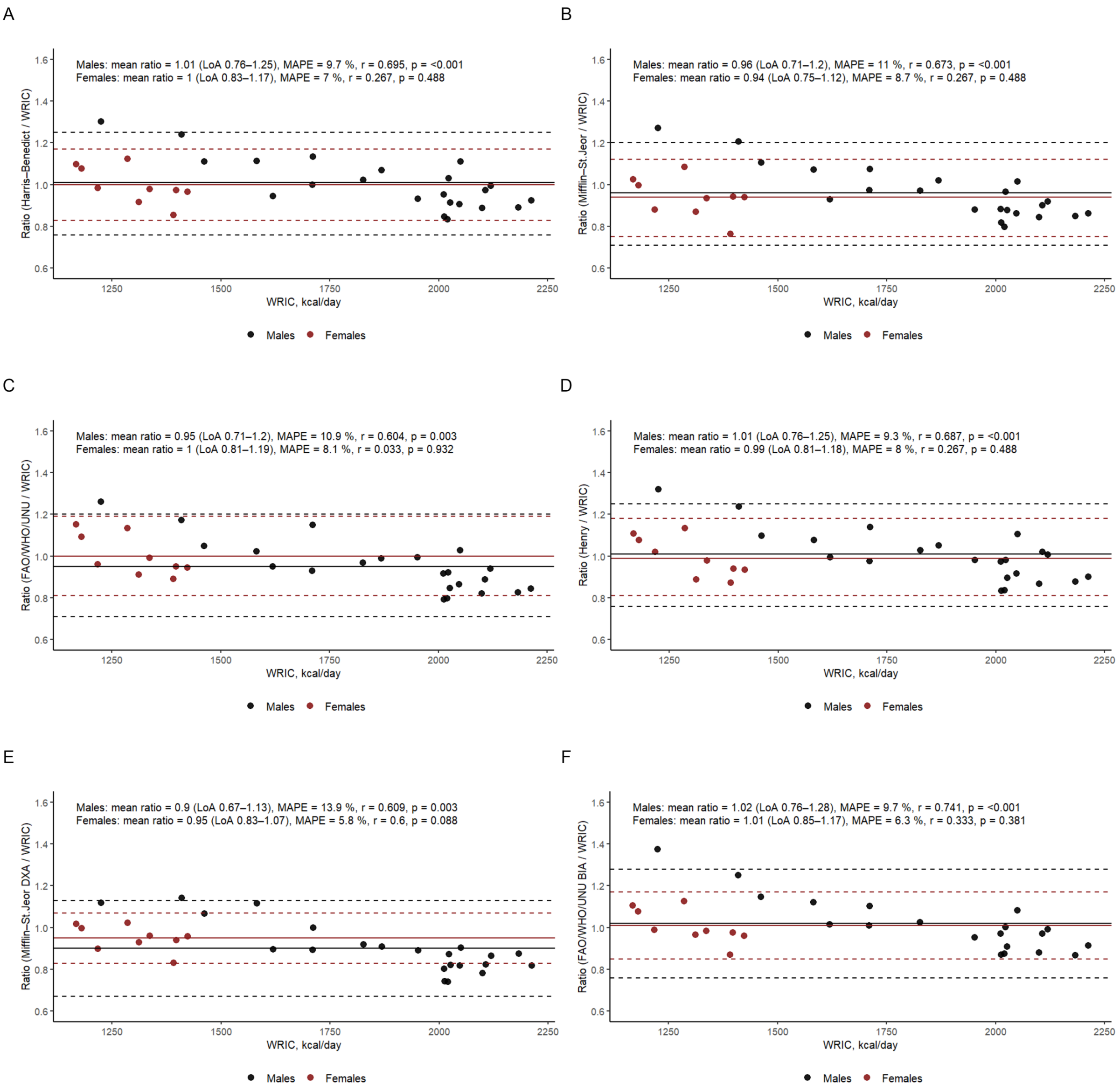

Supplementary Figure 1A–F: Sensitivity analysis. Bland-Altman plots showing the mean ratio between two methods for estimating REE against the criterion (measured REE) stratified by sex. Mean ratio, limits of agreement, MAPE, r- and p-values are presented separately for males and females. The REE was estimated in CRC survivors (n = 31; FAO/WHO/UNU$_{BIA}$, n = 29). **A:** Harris-Benedict vs. WRIC. **B:** Mifflin-St. Jeor vs. WRIC. **C:** FAO/WHO/UNU vs. WRIC. **D:** Henry vs. WRIC. **E:** Mifflin-St. Jeor$_{DXA}$ vs. WRIC. **F:** FAO/WHO/UNU$_{BIA}$ vs. WRIC. Abbreviations: BIA, bioelectrical impedance analysis; DXA, dual-energy x-ray absorptiometry; CRC; colorectal cancer; MAPE, mean absolute percentage error; p-value from correlation analysis presenting the significance of correlation; r, correlation coefficient form Spearman correlation analysis

showing relation between WRIC and the other methods; REE, resting energy expenditure; WRIC, whole-room indirect calorimetry.

Supplementary Figure 2A–F

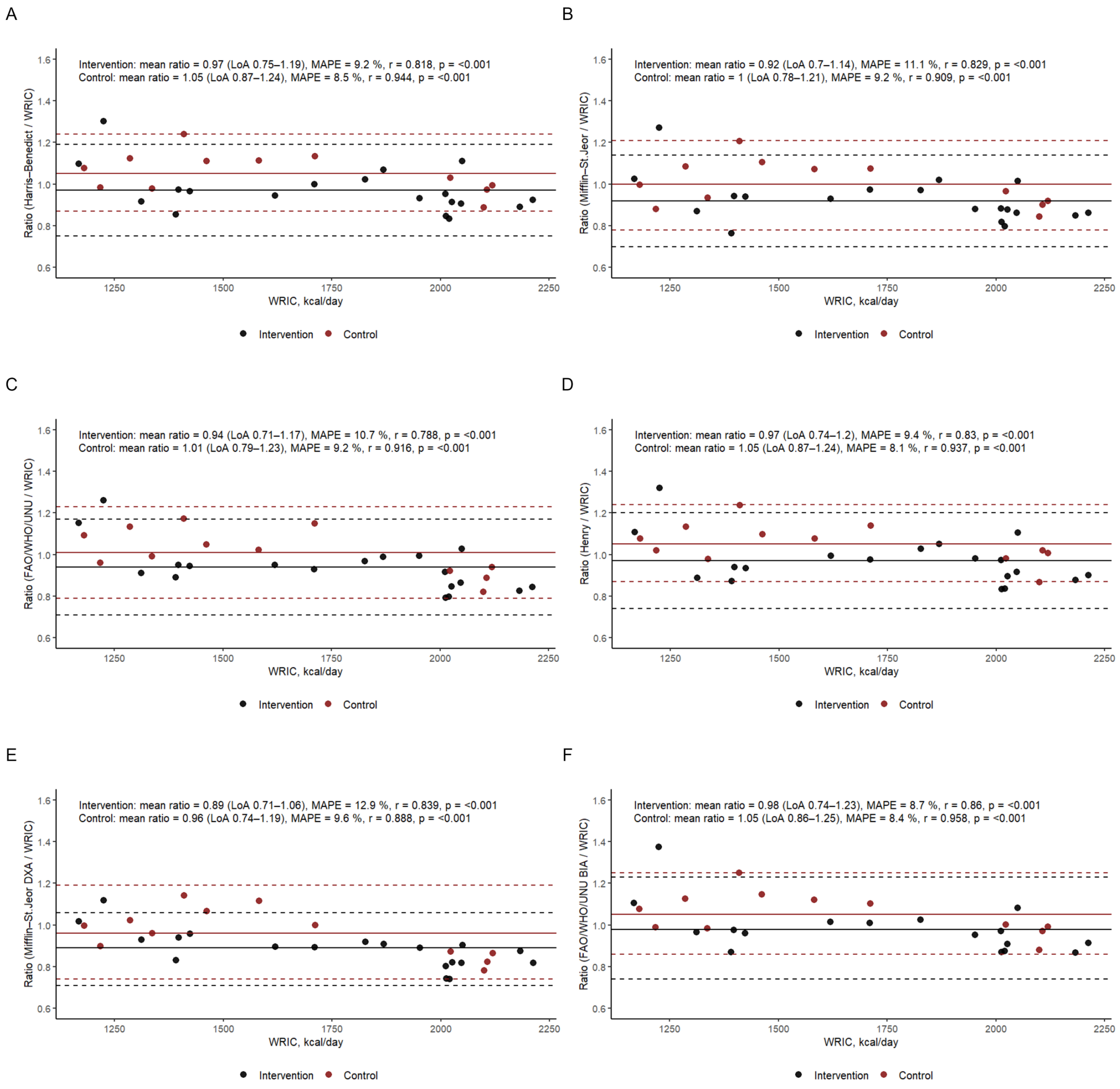

Supplementary Figure 2A–F: Sensitivity analysis. Bland-Altman plots showing the mean ratio between two methods for estimating REE against the criterion (measured REE) stratified by treatment group. Mean ratio, limits of agreement, MAPE, r- and p-values are presented separately for the intervention group and the control group. The REE was estimated in CRC survivors (n = 31; FAO/WHO/UNU$_{BIA}$, n = 29). **A:** Harris-Benedict vs. WRIC. **B:** Mifflin-St. Jeor vs. WRIC. **C:** FAO/WHO/UNU vs. WRIC. **D:** Henry vs. WRIC. **E:** Mifflin-St. Jeor$_{DXA}$ vs. WRIC. **F:** FAO/WHO/UNU$_{BIA}$ vs. WRIC. Abbreviations: BIA, bioelectrical impedance analysis; DXA, dual-energy x-ray absorptiometry; CRC; colorectal cancer; MAPE, mean absolute percentage error; p-value from correlation analysis presenting the significance of correlation; r, correlation coefficient form

Spearman correlation analysis showing relation between WRIC and the other methods; REE, resting energy expenditure; WRIC, whole-room indirect calorimetry.

Supplementary Figure 3A–F

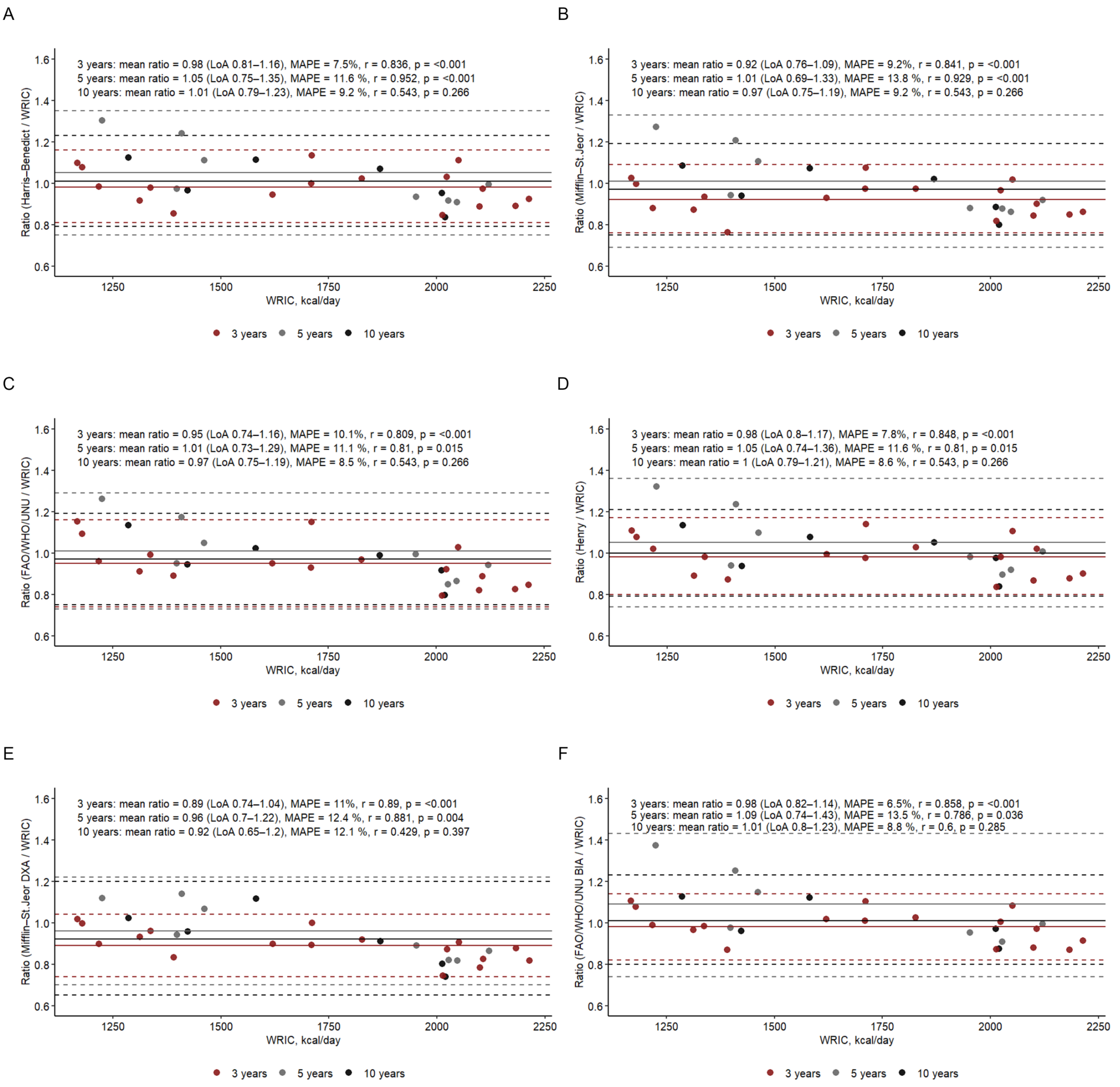

Supplementary Figure 3A–F: Sensitivity analysis. Bland-Altman plots showing the mean ratio between two methods for estimating REE against the criterion (measured REE) stratified by follow-up year. Mean ratio, limits of agreement, MAPE, r- and p-values are presented separately for three-, five- and, ten-year follow-up. The REE was estimated in CRC survivors (n = 31; FAO/WHO/UNU$_{BIA}$, n = 29). **A:** Harris-Benedict vs. WRIC. **B:** Mifflin-St. Jeor vs. WRIC. **C:** FAO/WHO/UNU vs. WRIC. **D:** Henry vs. WRIC. **E:** Mifflin-St. Jeor$_{DXA}$ vs. WRIC. **F:** FAO/WHO/UNU$_{BIA}$ vs. WRIC. Abbreviations: BIA, bioelectrical impedance analysis; DXA, dual-energy x-ray absorptiometry; CRC; colorectal cancer; MAPE, mean absolute percentage error; p-value from correlation analysis presenting the significance of correlation; r, correlation coefficient form Spearman

correlation analysis showing relation between WRIC and the other methods; REE, resting energy expenditure; WRIC, whole-room indirect calorimetry.

Supplementary Figure 4A–F

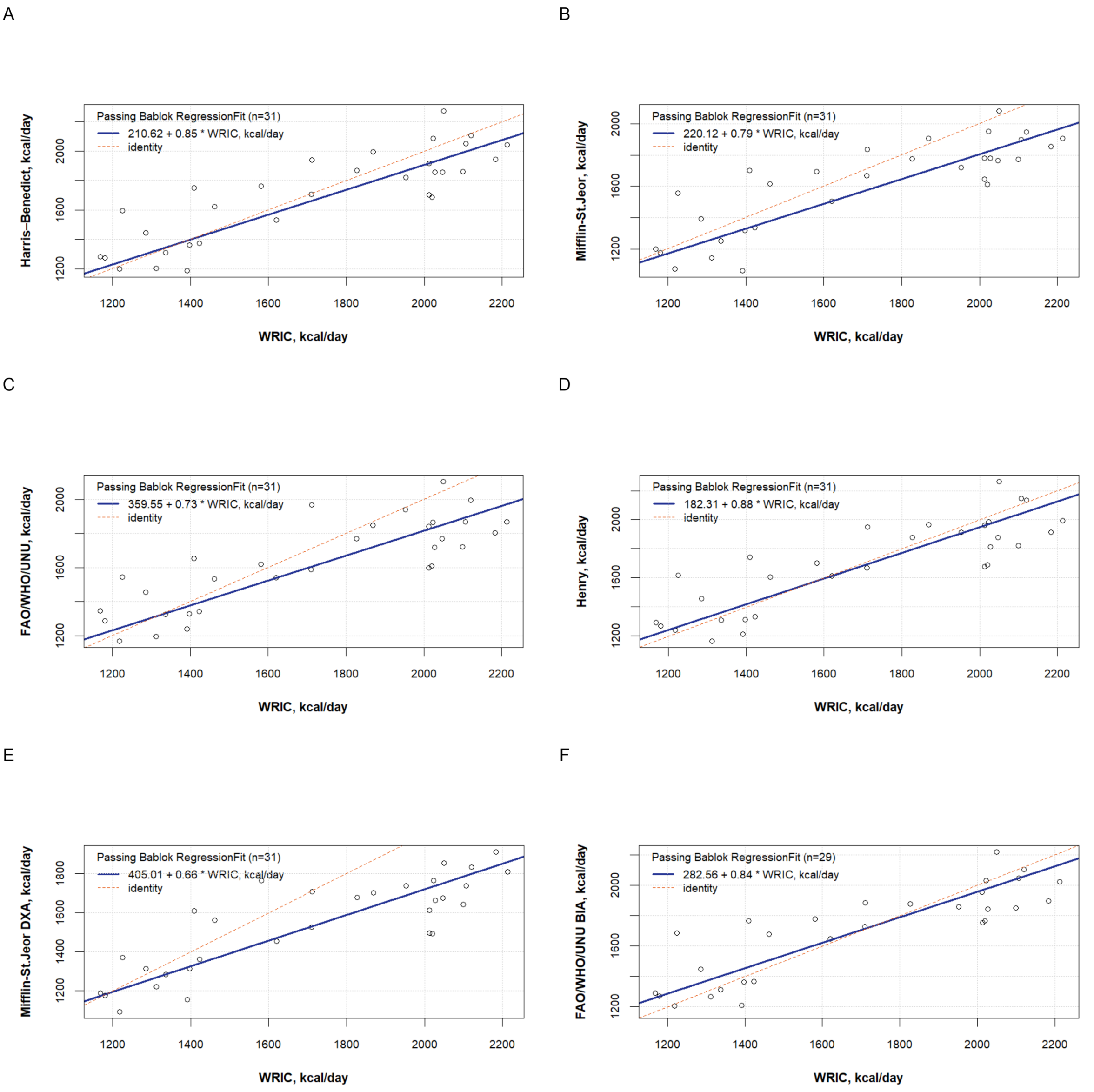

Supplementary Figure 4A–F: Passing–Bablok regression plots comparing REE from predictive equations and REE_{WRIC} among CRC survivors (n = 31; $FAO/WHO/UNU_{BIA}$, n = 29). The solid line and the dotted line show the regression line and line of identity (y = x), respectively. **A**: Harris-Benedict vs. WRIC. **B**: Mifflin-St. Jeor vs. WRIC. **C**: FAO/WHO/UNU vs. WRIC. **D**: Henry vs. WRIC. **E**: Mifflin-St. $Jeor_{DXA}$ vs. WRIC. **F:** $FAO/WHO/UNU_{BIA}$ vs. WRIC. Abbreviations: BIA, bioelectrical impedance analysis; CRC; colorectal cancer; DXA, dual-energy x-ray absorptiometry; REE, resting energy expenditure; WRIC, whole-room indirect calorimetry.

Supplementary Figure 5A–F

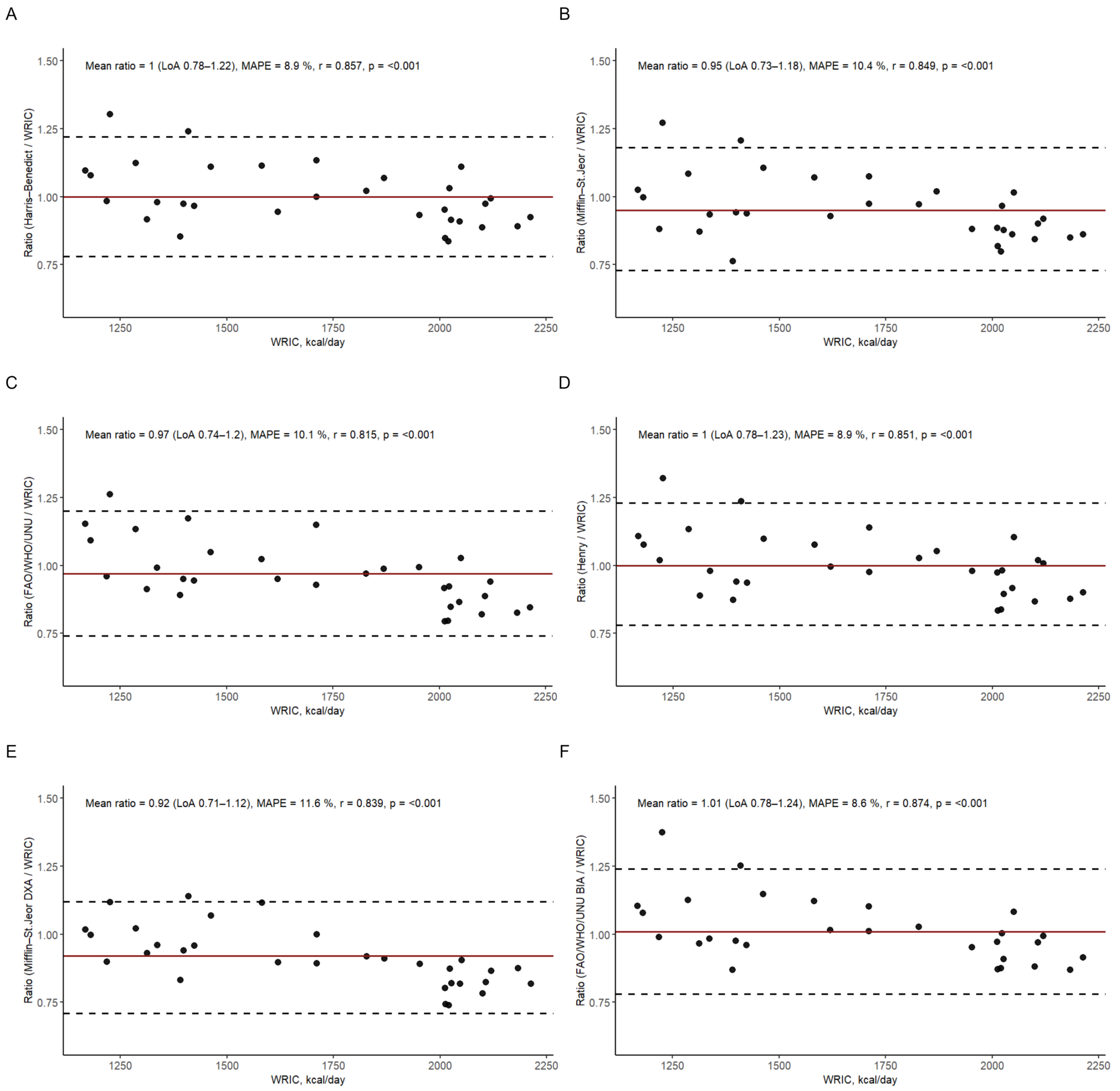

Supplementary Figure 5A–F: Sensitivity analysis, n = 22. Bland-Altman plots showing the mean ratio between two methods for estimating REE against the criterion (measured REE) after excluding subjects that did not adhere to the study protocol. In total, nine subjects were excluded from analysis (fasting, n = 2; nicotine, n = 2; caffeine, n = 2; strenuous activity, n = 2; nicotine and strenuous activity, n = 1). REE was estimated in CRC survivors. **A:** Harris-Benedict vs. WRIC. **B:** Mifflin-St. Jeor vs. WRIC. **C:** FAO/WHO/UNU vs. WRIC. **D:** Henry vs. WRIC. **E:** Mifflin-St. Jeor$_{DXA}$ vs. WRIC. **F:** FAO/WHO/UNU$_{BIA}$ vs. WRIC. Abbreviations: BIA, bioelectrical impedance analysis; CRC, colorectal cancer; DXA, dual-energy x-ray absorptiometry; MAPE, mean absolute percentage error; p-value from correlation analysis presenting the significance of correlation; r,

correlation coefficient form Spearman correlation analysis showing relation between WRIC and the other methods; REE, resting energy expenditure; WRIC, whole-room indirect calorimetry.
